# Use of Sugar Dispensers to Disrupt Ant Attendance and Improve Biological Control of Mealybugs in Vineyard

**DOI:** 10.3390/insects12040330

**Published:** 2021-04-07

**Authors:** Martina Parrilli, Marco Profeta, Luca Casoli, Fabio Gambirasio, Antonio Masetti, Giovanni Burgio

**Affiliations:** 1 Dipartimento di Scienze e Tecnologie Agro-Alimentari (DISTAL), Alma Mater Studiorum-Università di Bologna (UNIBO), Viale G. Fanin 42, 40127 Bologna, Italy; antonio.masetti@unibo.it (A.M.); giovanni.burgio@unibo.it (G.B.); 2 Consorzio Fitosanitario Provinciale di Reggio Emilia, via F. Gualerzi 32, 42124 Reggio Emilia, Italy; marco.profeta@regione.emilia-romagna.it (M.P.); luca.casoli@regione.emilia-romagna.it (L.C.); gambirasio.agro@gmail.com (F.G.)

**Keywords:** ant-mealybug association, sugar dispensers, *Planococcus ficus*, *Pseudococcus comstocki*, *Anagyrus vladimiri*, *Cryptolaemus montrouzieri*, biological control, vineyard

## Abstract

**Simple Summary:**

Management methods for mealybugs (Hemiptera: Pseudococcidae) alternative to insecticides have been explored in vineyards. Biological control by either wild or released natural enemies can be disrupted by tending ants, which create a strong association with mealybugs. In this paper, sugar dispensers were investigated as an ant management method to enhance parasitization and predation and eventually to reduce mealybug infestations. Field trials showed a reduction of ant activity, an enhancement of the ecosystem services provided by both parasitoids and predators and a decrease of mealybug infestation on grapes. The use of sugar dispensers provided promising results, highlighting its potential to be integrated with inoculative releases for a more sustainable management of mealybugs.

**Abstract:**

*Planococcus ficus* (Signoret) and *Pseudococcus comstocki* (Kuwana) (Hemiptera: Pseudococcidae) are economically important pests occurring in vineyards, causing severe economic losses for growers and compromising bunch production. The partial effectiveness of insecticides used in controlling mealybug infestations as well as their high impact on the environment and on human health have led to the research of alternative and sustainable control methods, including biological control. Several natural enemies are reported to be effective against mealybugs, but their activity may be hindered by tending ants. These social insects are known to exhibit a mutualistic relationship with mealybugs, resulting in extremely aggressive behavior against beneficial insects. Consequently, this study explored a method to mitigate ant attendance by means of sugar dispensers in order to improve ecosystem services, as well as decrease mealybug infestation in vineyards. Field trials were carried out in four commercial vineyards of Northern Italy infested by mealybugs, in which *Anagyrus vladimiri* Triapitsyn (Hymenoptera: Encyrtidae) and *Cryptolaemus montrouzieri* Mulsant (Coleoptera: Coccinellidae) were released as biological control agents. Our results showed that sugar dispensers reduced ant activity and mealybug infestation, leading to a significant enhancement of ecosystem services. The technique showed a great potential in boosting biological control against mealybugs in field conditions, though the field application seemed to be labour intensive and needs to be replicated for a multi-year evaluation.

## 1. Introduction

Mealybugs (Hemiptera: Pseudococcidae) are key pests of vineyards. Their feeding activity, as well as their excretion of large amount of honeydew, are responsible for severe damages on plants, especially on fruit production [1,2,3]. Pesticides still represent the most common strategy to control mealybugs. Nevertheless, the mealybug habit of staying in concealed plant parts and the waxy cover of these insects make chemical control in some conditions poorly effective [4]. New tactics, alternative to chemicals, show a potential to reduce and suppress mealybug infestations, including biological control [5]. Previous studies showed the potential of natural enemies, such as *Anagyrus vladimiri* Triapitsyn [3] and *Cryptolaemus montrouzieri* Mulsant, in controlling these detrimental pests [6,7]. However, few studies reporting evaluation of the field effectiveness of these biological agents are available. In Northern and Central Italy, several releases of *A. vladimiri* and *C. montrouzieri* have been carried out in the last few years and a field efficacy evaluation of these biological agents is in progress (Parrilli, unpublished data). Preliminary assessments of biological control programs reported a high effectiveness of inoculative releases [8], characterized by some degree of variability depending on geographic area, phytosanitary treatments of vineyard, and occurrence of attending ant (Hymenoptera: Formicidae) species (Parrilli, unpublished data). 

It is well known that the activity of biological control agents on mealybugs may be hindered by mealybug-tending ants which create a strong association with these hemipterans. Some ant species develop a mutualistic relationship with mealybugs due to their honeydew-consuming habit. These social insects feed on honeydew and, in return, mealybugs can get several benefits. Tending ants build earthen shelters to protect mealybugs from adverse weather conditions and prevent nymphs getting stuck in their honeydew [4]. Moreover, ants facilitate mealybug dispersal and provide them protection against natural enemies. Parasitoids and predators are often attacked by tending ants, which are particularly hostile to any possible harmful movement around a honeydew source [4]. Ant aggressiveness often disturbs natural enemy activity, thereby causing biological control disruption [9] or reduced effectiveness of the released beneficials. Mani and Shivaraju [4] showed several examples of natural enemy failures in controlling mealybugs due to the presence of ant attendants.

Ant aggressiveness depends on species and Buckley and Gullan [10] demonstrated that coccoids attended by relatively inoffensive ants were more parasitized than those attended by more aggressive species. Mgocheki and Addison [11] proved that the ant species *Anoplolepis steingroeveri* (Forel), *Crematogaster peringueyi* Emery and *Linepithema humile* (Mayr) significantly interfered with biological control of *Planococcus ficus* (Signoret). Also, *Tapinoma nigerrimum* (Nylander) was found to hinder and disrupt the activity of two main natural enemies of the vine and citrus mealybugs [12]. Fanani et al. [13] demonstrated that the parasitoid of cassava mealybug, *Anagyrus lopezi* (De Santis) (Hymenoptera: Encyrtidae), exhibited higher parasitism and emergence rates on ant-excluded plants compared to ant-attended plants in laboratory conditions.

Granular insecticides, exclusion methods, liquid baits, as well as sugar source provisioning have been already tested to control ants or mitigate their activity [14,15,16,17], showing promising results in reducing mealybug abundance and infestations [16,17,18,19]. Stanley [20] reviewed the efficacy of baits deployed for ant control and eradication. Liquid sucrose-based baits were particularly effective against tending ants, whose diet consists mainly of sugar [15,18]. Artificial sugar dispensers, with or without insecticides, have been tested [17,18]. The inclusion of insecticides can provide a control of ant population, whose members feed other colony individuals by trophallaxis. Insecticide addition may have detrimental effects on non-target insects, such as pollinators and natural enemies. Nevertheless, the small amount of pesticides and bait delivery system reduce undesirable effects compared to broad-spectrum insecticide sprays [18,21].

The impact of sugar dispensers on ecosystem services, such as parasitization and predation, has not been deeply evaluated in field conditions yet. Developing alternative sugar sources to reduce the population of mealybug tending ants may also help increasing natural enemy performance in vineyards. The goal of this work was to investigate if the use of sugar dispensers can reduce ant activity and attendance, thus enhancing the biological control against mealybugs, including the efficacy of the inoculative releases of *A. vladimiri* and *C. montrouzieri* in infested vineyards. Moreover, this study was aimed at describing ant assemblages in Northern Italy, an important area of grape cultivation, and evaluating the different level of protection to mealybugs that ant species foraging in vineyards can provide.

## 2. Materials and Methods

### 2.1. Sites and Experimental Plan 

The experiment was carried out in 4 vineyards infested by mealybugs, in Reggio Emilia Province, in 2020 (Table 1). Vineyards were selected based on the pest pressure recorded by extension services in recent years.

Conventional vineyards rely on synthetic chemical products for pest management, whereas multiple tactics (chemical, biological, agronomic, etc.) are used in Integrated Pest Management (IPM) vineyards. 

Inter-row ground cover vegetation was mowed close to the ground and no mealybug insecticides were applied in the trial areas.

The trial was carried out in a 0.6–1.5 ha area inside each vineyard. Two plots were selected within each area: sugar dispenser and control plots. Their sizes varied between 1200 and 2800 m^2^ and the minimum distance between plots was 20 m.

### 2.2. Sugar Dispensers

**Figure 1:**
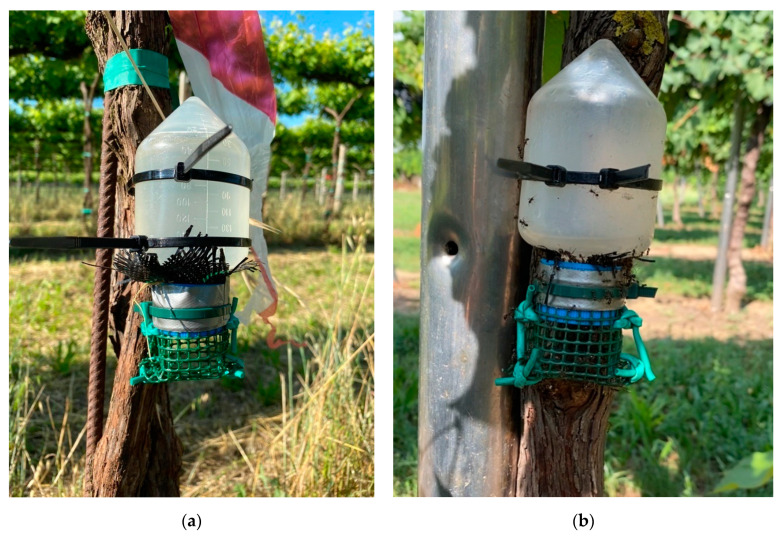
Sugar dispenser which was just refilled with sucrose liquid (**a**) and ants feeding on dispenser sucrose liquid (**b**).

Sugar dispensers (Figure 1) were built using a similar method to that of Daane et al. [18]. Falcon centrifuge tubes (175 mL) were used as sugar dispensers. A one-cm hole was drilled in each cap and 10 cm × 10 cm square of permeable plastic mesh was placed between the cap and tube. A plastic net (4 mm mesh) was placed on dispenser caps to avoid honeybee access to sucrose liquid.

Sugar dispensers were positioned on random vine trunks (40–50 cm above the ground) at the beginning of June and were removed at the beginning of September. Sugar dispensers were deployed at a density of about 120 dispensers/ha, evenly spaced throughout the sugar dispenser plot of each vineyard (Vineyard 1: 16 sugar dispensers; Vineyard 2: 15 sugar dispensers; Vineyard 3: 34 sugar dispensers; Vineyard 4: 30 sugar dispensers). Each sugar dispenser was filled with 25% sucrose aqueous solution and refilled fortnightly.

### 2.3. Natural Enemy Release

Natural enemies were released in order to ensure a comparable level of ecosystem services in each vineyard, mitigating as much as possible the variability of natural parasitization and predation among sites.

At the beginning of July (2nd and 8th of July), *A. vladimiri* was randomly released at a rate of 1.500 individuals/ha in two different moments to guarantee its permanence in the field. The first release included 1.000 wasps and, one week after, the other 500 individuals were released. Parasitoid releases were carried out after two weeks from mandatory treatment with tau-fluvalinate (MAVRIK 20 EW, Adama, Italy) against *Scaphoideus titanus* Ball. (Hemiptera: Cicadellidae). *Anagyrus vladimiri* releases were carried out near vine plants while walking along vineyard rows. The host-seeking activity of this parasitoid allows the release even when mealybug infestation is not clearly visible in field. 

The predator *C. montrouzieri* was released at a minimum density of 300 individuals/ha in all the sites starting at the end of July (29th of July), except in vineyard 1. In this vineyard, three consecutive releases (8th and 29th of July and 5th of August) of 300 *C. montrouzieri* individuals (for a total of 900 individuals) were carried out, due to the presence of early and severe mealybug infestation. In vineyard 4, two consecutive releases (29th of July and 5th of August) of 300 individuals/ha were performed to improve control of mealybug infestation, considering unexpected colony appearance in the previous years. *Cryptolaemus montrouzieri* releases were targeted on plants with high mealybug infestation. Honeydew and wax secretions are fundamental to stimulate the predator oviposition [22], so a high prey density is needed to ensure *C. montrouzieri* permanence in field.

Both *A. vladimiri* and *C. montrouzieri* were supplied by Bioplanet (Cesena, Italy), with which releases of natural enemies were set.

### 2.4. Ant Activity

Ant activity was estimated fortnightly by counting the number of ants crossing an imaginary line placed on vine trunk (at about 15–20 cm below the vine canopy) during a 1-min period [17]. The imaginary line was a transect whose length was equal to vine trunk diameter. In sugar dispenser plots, ant activity assessment was carried out on each vine on which sugar dispensers were hung, with the imaginary lines placed approximately 30 cm above sugar dispensers. It was expected to detect less ant individuals crossing the imaginary line because of the presence of sugar dispensers. Vines for ant counting in the controls were randomly selected to evenly represent the whole plot.

Samples of ants were collected fortnightly during the experiment from canopy and branches or collecting insects from sugar dispensers, in order to identify the species. Ant individuals were killed in a refrigerator and stored in test tubes (70% ethanol) until identification, which was accomplished by means of identification keys [23]. Relative abundance of each ant species was calculated as the ratio between the number of each ant species and the total number of ants collected. 

### 2.5. Infestation, Parasitization and Predation Sampling

Bunches were collected between the end of August and early September, just before harvest. 

To evaluate mealybug infestation in sugar dispenser plots, one randomly selected bunch was collected on each plant where dispensers were placed, for a total of 15–34 bunches/plot. Exactly the same number of bunches was collected randomly in control plots picking one bunch per vine on the same plants where ant activity was estimated.

To more precisely estimate parasitization and predation, 6–10 infested bunches (hereafter also referred as colonies) per plot were actively searched and collected. Vines where ant activity was estimated were excluded by the picking of infested bunches. Besides assessing parasitization on infested bunches, parasitized mealybugs were also estimated on randomly selected bunches (used to determine infestation) in order to evaluate the parasitoid activity at different density of mealybug infestation.

Infestation was assessed in two different ways, estimating bunch infestation rate and counting the number of mealybugs per bunch. In particular, the percentage of infested bunches was evaluated by a visual sampling in the field, counting the bunches with sign of infestation such as honeydew, sooty mould and mealybug presence. Once infested bunch rate was assessed, the same bunches were taken to the laboratory (Department of Agricultural and Food Sciences, University of Bologna), and the number of mealybugs was assessed counting individuals of each development stage (nymphs, adults (females), mature females) and distinguishing mealybug species, when possible. Parasitization was estimated as the ratio between parasitized mealybugs and the total number of mealybugs, considering only adult and mature females as they are the most suitable stages for *A. vladimiri*. Also, parasitization of nymph stage can lead to strong underestimation due the lack of certain symptoms of parasitoid attack. Presence of a single hole in the back of mealybug or swollen pest body were considered sign of parasitization. 

Lacerated bodies and eggs without mature adults were considered as sign of predation, so the ratio between predated mealybugs and the total number of mealybugs was used to calculate the percentage of predation. Finally, mean number of *C. montrouzieri* larvae per bunch was recorded as well.

### 2.6. Statistical Analysis

The mean number of ants counted on vine trunks was analyzed using a generalized linear mixed model (GLMM) with normal probability distribution and identity log-link function. Treatment (sugar dispenser and control) was included as fixed factor and sampling dates as repeated measures. Vineyards were considered as random block factor. Restricted maximum likelihood with Kenward-Roger’s approximation of the degrees of freedom (df) was selected.

A correspondence analysis was performed in order to correlate ant species with vineyards and to better describe potential variations in ant communities in the investigated sites.

Log linear analysis was used to analyse the average ratio of damaged bunches, parasitization on randomly-collected bunches and colonies, and predation on colonies. Log-linear analysis allows for simultaneous evaluation of multiple interactions among categorical variables, using a method that resembles a factorial analysis of variance [24]. Here, the response variables were the percentage of infested bunches, parasitization and predation, whereas the independent variables were treatment (sugar dispenser and control) and vineyards (*n* = 4). In the results, both the partial association and the marginal association tests were shown.

In each single vineyard, the effect of treatment on damaged bunches, parasitization and predation was evaluated by chi square test (χ^2^).

The software IBM SPSS Statistics (ver. 26) (IBM corporation, Armonk, NY, USA) and Statistica version 10 (StatsoftTM, Tulsa, OK, USA) were used for the analyses. 

## 3. Results

### 3.1. Ant Species

**Figure 2:**
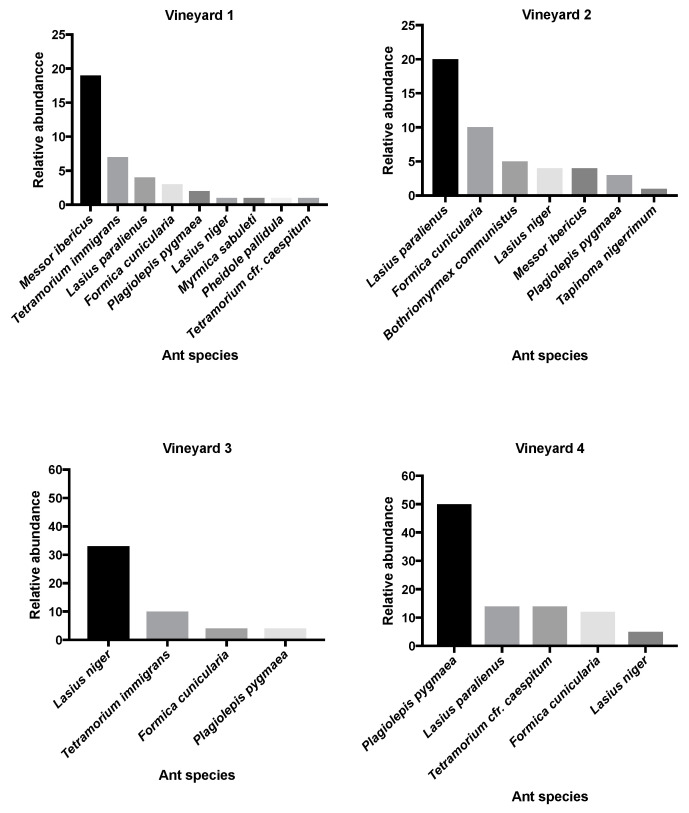
Relative abundance (%) of ant species collected in each vineyard. Relative abundance was calculated as the ratio between the number of each ant species and the total number of ants collected.

**Figure 3:**
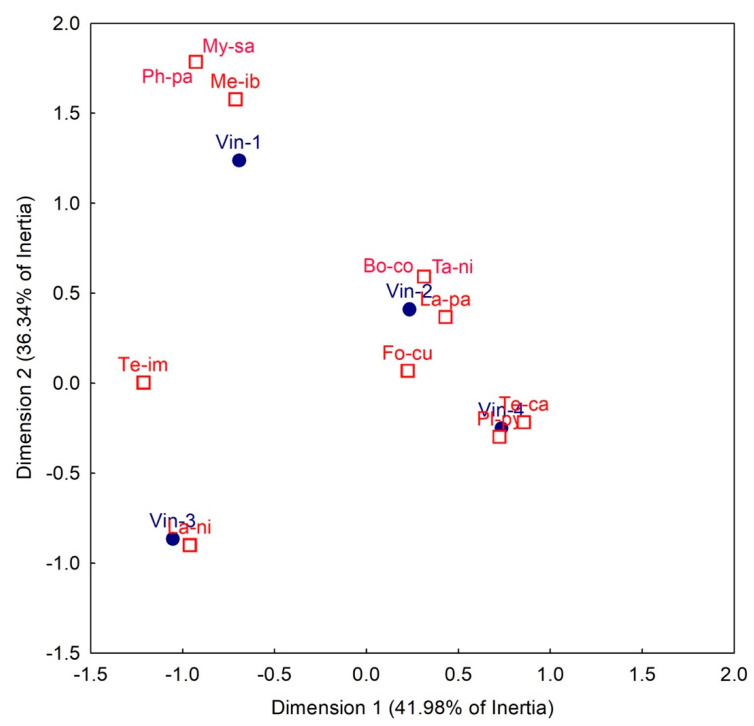
Biplot of the two first axes of the Correspondence Analysis relating ant species (represented by open red squares) and vineyards (represented by blue circles). Ant species: My-sa = *Myrmica sabuleti*; Ph-pa = *Pheidole pallidula*; Me-ib = *Messor ibericus*; Bo-co = *Bothriomyrmex communistus*; Ta-ni = *Tapinoma nigerrimum*; La-pa = *Lasius paralienus*; Fo-cu = *Formica cunicularia*; Te-im = *Tetramorium immigrans*; Te-ca = *Tetramorium cfr. caespitum*; Pl-py = *Plagiolepis pygmaea*; La-ni = *Lasius niger*. Vineyards: Vin-1 = vineyard 1; Vin-2 = vineyard 2; Vin-3 = vineyard 3; Vin-4 = vineyard 4.

Overall, 11 ant species were recorded in field sites (Figure 2), for a total of 232 ant individuals collected. The highest number of species (9) was found in vineyard 1, whereas the lowest number of ant species (4) was observed in vineyard 3, that was characterized by the dominance of the aggressive *Lasius niger* (L.). Most of the ant species collected display a sugar feeding behavior and only the genus *Messor* consists of seed harvesting ants [25]. As integration of Figure 2, Figure 3 provides a statistic support of dominant ant species in each site, explaining near the 80% of inertia.

### 3.2. Ant Activity

**Figure 4:**
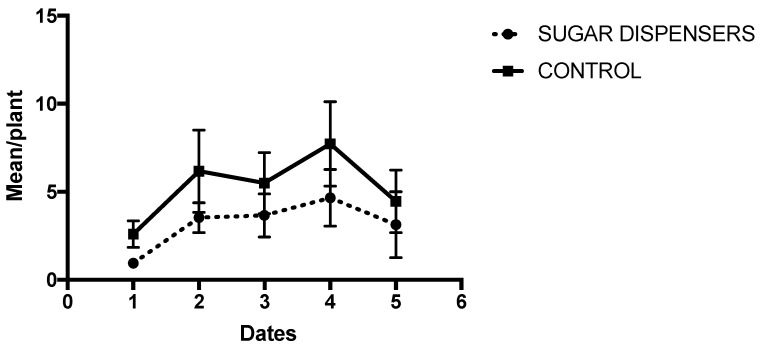
Average ant activity (± SE) in sugar dispenser and control treatments during summer 2020 (*n* = 4). The sampling period started on 17th–19th of June (Date 1) and ended on 26th–28th of August (Date 5). Ant activity was estimated fortnightly. GLMM showed a significant effect of sugar dispensers on ant activity (*p* = 0.034), as well as a significant effect of sampling dates (*p* = 0.017).

Figure 4 shows the average ant activity during the summer 2020. In all monitoring dates, the mean ant activity was significantly lower in sugar dispenser plots compared to that of controls. A significant effect of sampling dates on ant activity was also detected (Table 2). GLMM did not detect any significant effect of the vineyards, which were included in the statistical analysis as a random block factor (Z = 0.74; *p* = 0.46).

**Figure 5:**
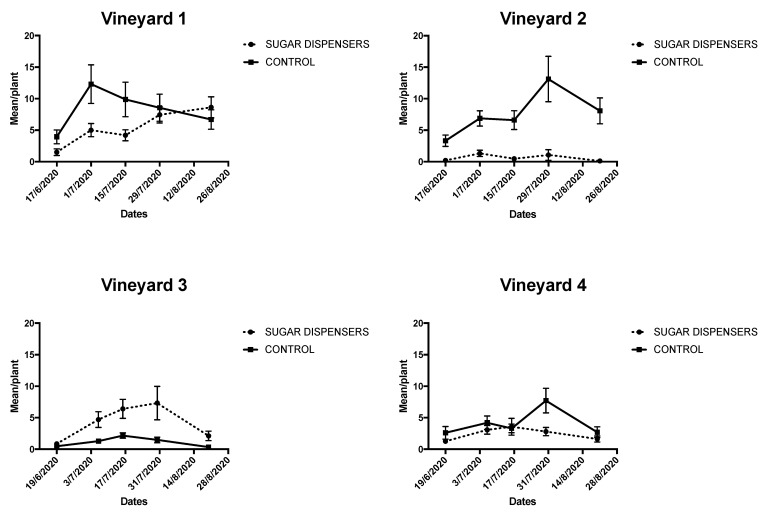
Ant activity (± SE) in sugar dispenser and control plots in each vineyard. Ant activity was estimated by counting the number of ants crossing an imaginary line on vine trunk in 1-minute period.

A trend of ant counting on vines is also reported separately for each vineyard in order to better evaluate the behaviour of ants (Figure 5). Vineyard 1 was characterized by higher ant activity in control than sugar dispenser treatment in 3 dates out of 5. A similar mean number of tending ants was counted in the two treatments in the last monitoring dates (Figure 5). Lower ant activity in sugar dispenser treatment compared to control plot was detected in vineyard 2 during the entire season (Figure 5), with strong differences between the two treatments. On the other hand, more tending ants were counted in sugar dispenser treatment than control in four out of five sampling dates in vineyard 3 (Figure 5). Finally, in vineyard 4 ant activity was lower in the sugar dispenser plot than control, apart from the third date (Figure 5).

### 3.3. Mealybug Infestation

**Figure 6:**
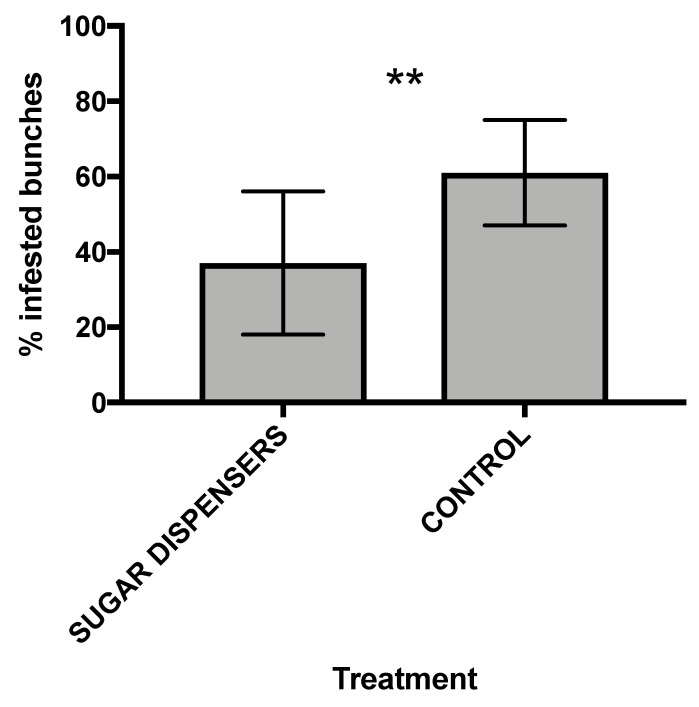
Mean percentages of infested bunches (± SE) (*n* = 4). Log linear analysis showed a significant difference between the two treatments (** = *p* < 0.01).

The use of sugar dispensers significantly reduced the percentage of infested bunches in dispenser treatment compared to control (*p* < 0.01); partial association test showed the same result of marginal association one (Table 3, Figure 6). A vineyard effect was also observed on the percentage of infested bunches (Table 3). On the other hand, the mean number of mealybugs per bunch registered by random sampling was similar in control and sugar dispenser plots (sugar dispensers: 172.64 ± 164.16 (SE); control: 189.55 ± 170.98 (SE)).

A further analysis of bunch infestation was carried out in each vineyard, in order to better explain the infestation dynamics in each site. Two vineyards out of 4 had a significantly higher percentage of infested bunches in control plot compared to sugar dispenser plots (Figure S1). In vineyard 2, 13% of bunches were infested by mealybugs in the sugar dispenser treatment, whereas in control the infestation level was 73% (df = 1; χ^2^ = 11; *p* < 0.001) (Figure S1). Also vineyard 4 showed a significantly lower infestation in the sugar dispenser treatment (17%) than in control (43%) (df = 1; χ^2^ = 5.08; *p* = 0.02) (Figure S1). On the other hand, approximately the same level of infestation was found in vineyard 1 and 3 in both treatments (Figure S1). 

These infestation patterns are also confirmed by the mean number of mealybugs per bunch. Indeed, 3.73 ± 2.66 mealybugs per bunch were found in the sugar dispenser treatment, while 18.80 ± 5.35 mealybugs per bunch were counted in control plot of vineyard 2. Also, vineyard 4 presented a higher number of mealybugs per bunch in the control treatment (17.73 ± 5.70) compared to the dispenser one (3.43 ± 2.51). A similar number of mealybugs per bunch was observed in both treatments of vineyard 1 and 3. 

### 3.4. Parasitization and Predation

**Figure 7:**
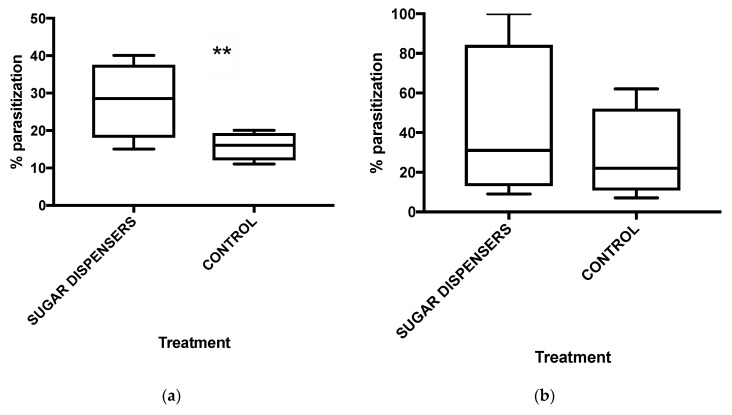
Mean percentages of parasitization (*n* = 4) on colonies (**a**) and randomly-collected bunches (**b**). Box plots indicate the median (solid line) and the range of dispersion (the lower and upper quartiles); the whiskers (vertical lines) represent the minimum and maximum parasitization rates observed. Log linear analysis detected a significant difference of parasitization rate between the two treatments on colonies (** = *p* < 0.01) (**a**), whereas no significant difference of parasitization rate was observed between sugar dispenser and control plots on randomly-collected bunches (*p* > 0.05) (**b**).

Log linear analysis showed a significantly higher parasitization rate in colonies from sugar dispenser treatment (Table 4, Figure 7a), whereas no significant difference was observed on parasitisation detected in randomly-collected bunches (Table 5, Figure 7b). Also, a significant effect of vineyard was detected on randomly-collected bunch parasitization (Table 5).

Regarding colony parasitization, only vineyard 4 presented significantly more parasitized mealybugs in dispenser treatment compared to the control one (df = 1; χ^2^ = 14.32; *p* < 0.001) (Figure S2). The parasitized mealybugs were twofold higher in sugar dispenser plot in comparison with that of control in vineyard 2 (Figure S2), but this difference was not supported by chi-square test. A level of parasitization of 27% was observed in sugar dispenser treatment, while just 20% of parasitized mealybugs were found in control plot in vineyard 3 (Figure S2). Vineyard 1 presented about the same percentage of parasitized mealybugs in the two treatments (Figure S2).

Parasitization rate in randomly-collected bunches was higher in sugar dispenser plot than in control plot in vineyard 1 (Figure S3), even if this difference was not supported by chi-square test. The percentage of parasitized mealybugs was slightly higher in sugar dispenser treatments compared to controls on randomly-collected bunches of vineyards 3 and 4 (Figure S3). On the other hand, in vineyard 2, a 100% of parasitization was found in the sugar dispenser plot, whereas 62% of parasitized mealybugs were observed in the control plot (Figure S3). 

**Figure 8:**
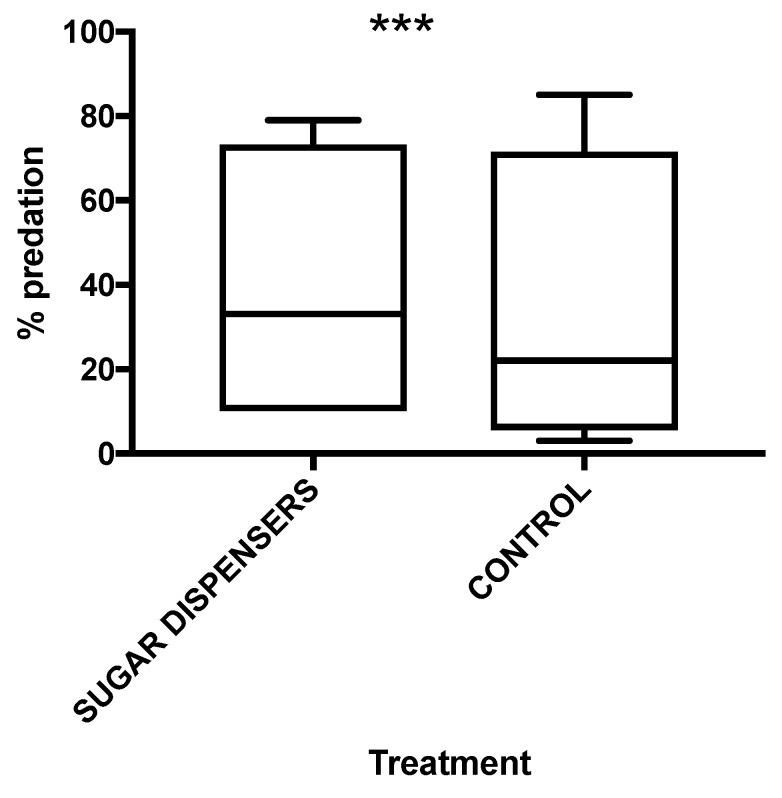
Mean percentages of predation (*n* = 4) in sugar dispenser and control treatments. Box plots indicate the median (solid line) and the range of dispersion (the lower and upper quartiles); the whiskers (vertical lines) represent the minimum and maximum predation rates observed. Log linear analysis showed a significant difference of predation rate between the two treatments (*** = *p* < 0.001).

Concerning *C. montrouzieri*, predated mealybugs were more frequently recorded among colonies in sugar dispenser treatment compared to control (Table 6, Figure 8) (*p* < 0.001). The greater activity of predators, most of which likely belonged to *C. montrouzieri,* was also confirmed by the mean number of *C. montrouzieri* larvae per bunch (*n* = 2), which was higher in sugar dispenser colonies (0.44 ± 0.27) than control ones (0.30 ± 0.20). Finally, a significant effect of vineyard on the percentage of predated mealybugs was recorded (*p* < 0.001) (Table 6).

The percentage of predated mealybugs on colonies was very high both in sugar dispenser and control treatments in vineyard 1, even if it was slightly higher in control plot (Figure S4). This evidence agrees with the higher density of the predator larvae per bunch in control (0.50 ± 0.50) compared to sucrose dispenser plot (0.17 ± 0.17). Vineyard 2 showed a similar level of predated mealybugs in colonies both in the sugar dispenser plot and the control one (Figure S4). Moreover, no *C. montrouzieri* larvae were collected in both treatments in vineyard 2. Colony predation was significantly lower in the control compared to sugar dispenser plot in vineyard 3 (χ^2^ = 12.51; df = 1; *p* < 0.001) (Figure S4), but no predator larvae were found during sampling at harvest. Finally, the percentage of predated mealybugs was significantly higher in the sugar dispenser plot than control in vineyard 4 (χ^2^ = 6.23; df = 1; *p* = 0.01) (Figure S4); indeed, more *C. montrouzieri* larvae were collected where sugar dispensers were present (0.70 ± 0.15), compared to control plot (0.10 ± 0.10).

## 4. Discussion

The use of liquid sucrose dispensers significantly enhanced ecosystem services in vineyards. This outcome is likely attributable to a reduction of ant activity, which was significantly lower in the presence of sugar dispensers. The percentage of infested bunches was significantly lower in sugar dispenser treatments compared to control plots. Also Beltrà et al. [17] demonstrated that the provisioning of sugar dispensers reduced vineyard infestation in terms of vine mealybug abundance. The use of sugar dispensers with insecticides decreased fruit damage also in California vineyards [18]. Despite a reduction of percentage of infested bunches in plot with sugar dispensers, the mean number of mealybugs per bunch detected by random bunch sampling was similar in both treatments. This result seems to demonstrate that the provisioning of sugar dispensers acts mostly in decreasing mealybug spatial diffusion in the field, for example reducing colony formation, than reducing colony size. Anyhow, the very high infestation of vineyard 1 contributed to the level of the mean mealybug infestation in both treatments; notwithstanding, a lower population in the sugar dispenser plot in comparison with control was recorded in two vineyards out of four. The decrement in the number of colonies would favour biological approaches for mealybug suppression, for example releasing *C. montrouzieri* only on plants where colonies occur, providing a more precise and effective control. Moreover, at least in two vineyards out of four, it seems that a relationship between mealybug abundance and infested bunch rate was present. Growers and consultants could benefit from this relationship and use the number of infested bunches instead of counting the number of mealybugs as a decision-making tool to define the severity of mealybug infestation.

Regarding ecosystem services, a significantly higher colony parasitisation in sugar dispenser plots in comparison with controls was recorded. Similarly, Pérez-Rodríguez et al. [26] found higher *Planococcus citri* (Risso) parasitism in citrus trees with sugar-feeders on the branches compared to control trees. In our trial, a tendency of higher mealybug parasitization was also found in dispenser treatment on randomly-collected bunches, but this difference was not significant. These different responses in parasitisation according to the types of bunch sampling (randomly-collected bunches *vs* colonies) were likely caused by the size of mealybug samples and ant behaviour. The higher number of mealybugs in colony than in randomly-collected bunches contributed to a more robust evaluation of this ecosystem service, leading to the significant effect of the treatments on colony parasitisation. Moreover, greater mealybug aggregation attracts more tending ants, which can benefit from higher amounts of honeydew. Sugar dispensers may “distract” ants more effectively from colonies, making mealybugs more susceptible to natural enemies. Also *C. montrouzieri* benefitted by the reduction of ant visits on colonies; indeed, average predation rate was significantly higher in sugar dispenser plots compared to control ones. 

Apart from enhancing beneficial activity, reduced ant-attendance might also have caused accumulation of honeydew on mealybug bodies, which could lead to higher mortality, especially of first instar nymphs [4,17].

A high variability was detected among vineyards both in terms of infestation and ecosystem services. Overall, the use of sugar dispensers reduced ant activity in most of the vineyards. Only vineyard 3 presented an inverted trend in some sampling dates. This outcome may be justified by an inhomogeneous mealybug density between treatment plots due to high spatial aggregation of this pest. Indeed, low infestation was found in the control plot of vineyard 3 during ant activity monitoring, whereas more mealybugs and tending ants were counted in the sugar dispenser area. Nevertheless, there was no significant difference between the percentage of infested bunches in dispenser and control plots before harvest. Sugar dispensers likely decreased ant-attendance thus leading to a non-significant difference in damaged bunches between the two treatments at the end of the season. 

Concerning parasitization and predation, *A*. *vladimiri* and *C. montrouzieri* showed a complementary action, which was enhanced by provisioning sugar dispensers. The highest parasitization rates were observed in vineyard 2 and 4, where bunch damages were caused principally by *P. ficus*. Anyhow, in our trial, a significant parasitization on *P. comstocki* was recorded. Our results are in agreement with a recent study reporting that *A. vladimiri* successfully parasitized both *P. ficus* and *P. comstocki* [3]. The highest predation pressures on mealybugs were detected in vineyard 1 and 3, which were infested by *P. comstocki*. The high abundance of *C. montrouzieri* larvae in the control plot of vineyard 1 was likely due to the high mealybug density in the control plot, thereby confirming the strongly density-dependent behaviour of the predator. Overall, the use of sugar dispensers showed a tendency to increase biological control in each field site, in terms of parasitization or predation rate.

Parasitized mealybugs may have been overestimated since only adult and mature females were used to calculate parasitization rate. On the other hand, if nymphal stages had been considered, parasitized mealybugs would have been underestimated, disguising *A. vladimiri* potential in controlling mealybug population. Before harvest, bunches are primarily infested by juvenile stages, on which it is extremely demanding to visually distinguish their three instars and detect parasitization signs.

The performances of natural enemies of mealybugs as well as their infestations were likely influenced by ant species. A high diversity of ants was observed in this trial, highlighting clear differences in species assemblages among vineyards. This underlines how different the disrupting activity of ant individuals against natural enemies could be, depending on their behavioural characteristics and species. The highest number of species was found in vineyard 1; three of them (*L. niger*, *Tetramorium immigrans* Santschi and *Tetramorium* cfr. *caespitum* (Linnaeus)) are considered very aggressive [27]. The most abundant ant species in vineyard 1 was *Messor ibericus* Santschi. Species belonging to the genus *Messor* have been already found in vineyards, even if this genus encompasses mainly seed harvester ants [28]. *Lasius niger* was also the most abundant species recorded in vineyard 3. This ant is known for protecting *P. comstocki* mealybugs, building shelters made by earth grains [29]. *Lasius paralienus* Seifert and *Plagiolepis pygmaea* (Latreille) were the most abundant ant species in vineyard 2 and 4, respectively. The genus *Plagiolepis* was already found foraging on vines, by Beltrà et al. [17]. Both *L. paralienus* and *P. pygmaea* species are considered less aggressive than the species found in vineyard 1 and 3; thus, it would seem that colony parasitisation and sugar dispenser efficacy were higher in the vineyards attended by these less aggressive ants (vineyard 2 and 4). Moreover, the coexistence of several aggressive ant species in the same site may have amplified their disrupting activity against released beneficials. Sugar dispensers may not have been able to compensate ant-attendance and consequently enhance natural enemy performance. Overall, sugar dispensers may be more effective in vineyards characterized by more harmless ants compared to those where aggressive ants are common. 

Just a few individuals of *T. nigerrimum* were detected in our study although this ant species has been reported as one of the most common ant species associated with vine and citrus mealybugs in the Mediterranean areas [12].

Sugar dispenser density of this experiment (about 120/ha) provided a reduction of tending-ant population. Nelson and Daane [30] showed that in their experiment there was not an optimal ant dispenser density which maximised ant population control. Although, they suggested that deploying more dispensers could provide higher ant and mealybug suppression. Moreover, in order to maximize the impact of this tactic on ant population, dispensers should be positioned in the field starting from spring, as Nelson and Daane [30] pointed out.

Insecticides, such as boric acid or neonicotinoids, may be added to sucrose liquid. The addition of pesticides could provide a suppression of ant population, acting on ant brood which are usually present in spring. However, the use of insecticides within sucrose solution should be suspended when inoculative releases of natural enemies are carried out and during flowering of ground cover plants, which could be intensively visited by pollinators. In this way, side effects on pollinators and other beneficials might be avoided, even if Cooper et al. [31] and Tay et al. [21] concluded that the low quantity of insecticide deployed in ant baits should have a small impact on non-target insects. However, the use of pesticide in sugar dispenser seems to pose some risks that should be avoided for a true ecological management of vineyard. Furthermore, insecticides should be legally authorized for this particular use.

Dispenser provisioning should be adopted continuously for some consecutive years to optimize the efficacy of sugar dispensers against ant population. There is evidence that ant activity and also mealybug infestations were reduced more strongly in the second year of a bait program, especially when ant populations were high [30]. 

Future trials should also focus on figuring out alternative delivering methods of sucrose liquids. Installation and maintenance of the sugar dispensers described in our experiment are labour-intensive to be adopted by growers. Recently, new methods have been studied to overcome conventional liquid baiting drawbacks. For example, Tay et al. [21] demonstrated that alginate hydrogel provided an efficient delivery system for liquid baits to control Argentine ant *L. humile*.

## 5. Conclusions

In conclusion, this field trial confirms the potential efficacy of sugar provision to reduce ant activity, as other studies already reported [17,32]. Moreover, this work proves that managing ant-attendance can enhance biological control provided by *A. vladimiri* and *C. montrouzieri*. Several studies showed examples of ant deterring parasitoid and predators of mealybugs, as reviewed by Mani & Shivaraju [4]; this experiment quantifies for the first time the impact of sugar dispensers on released natural enemy ecosystem services, such as parasitization and predation, in vineyard field conditions. This tactic, if confirmed by a multi-year evaluation and in variable condition scenarios, could be adopted within mealybug management. Thus, ant attendance disruption could be integrated with inoculative releases of beneficials in vineyards to boost natural enemy activity in a sustainable and effective way.

## Figures and Tables

**Table 1:** Details of the vineyard sites used for the field trial.

Site	Province	Longitude	Latitude	Variety	Training System	Mealybug Species	Pest Management
1	Reggio nell’Emilia	10°48’28.38” E	44°47’22.71” N	Lambrusco Salamino	Espalier	*P. comstocki*	Conventional
2	Reggio nell’Emilia	10°44’50.15” E	44°47’29.55” N	Ancellotta	Géneve Double Courtain (GDC); Espalier	*P. ficus* and *P. comstocki*	IPM
3	Reggio nell’Emilia	10°43’54.41” E	44°51’36.36” N	Lambrusco Marani	Espalier	*P. comstocki*	IPM
4	Reggio nell’Emilia	10°43’29.18” E	44°50’01.45” N	Lambrusco Marani	Espalier	*P. ficus* and *P. comstocki*	Conventional

**Table 2:** Factor effects in the generalized linear mixed model carried out on mean number of ants counted on the vines.

Factor	F	df1	df2	*p*
Treatment	5.15	1	21.33	0.034
Date	4.94	4	10.44	0.017
Treatment x date	0.12	4	10.44	0.973

**Table 3:** Log linear results showing the effect of each factor (T = treatment; V = vineyard) on the percentage of infested bunches (I = infestation).

Effect	df	Chi Square (Partial Association Test)	*p* (Partial Association Test)	Chi Square (Marginal Association Test)	*p* (Marginal Association Test)
T × I	1	10.70	0.001	8.33	0.004
V × I	3	47.96	<0.001	45.60	<0.001

**Table 4:** Log linear results showing the effect of each factor (T = treatment; V = vineyard) on colony parasitization rate (PaC = parasitization rate on colonies).

Effect	df	Chi Square (Partial Association Test)	*p* (Partial Association Test)	Chi Square (Marginal Association Test)	*p* (Marginal Association Test)
T × PaC	1	11.46	<0.001	10.68	0.001
V × PaC	3	7.46	0.06	6.67	0.08

**Table 5:** Log linear results showing the effect of each factor (T = treatment; V = vineyard) on parasitization rate on randomly-collected bunches (PaR = parasitization rate on randomly-collected bunches).

Effect	df	Chi Square (Partial Association Test)	*p* (Partial association Test)	Chi Square (Marginal Association Test)	*p* (Marginal Association Test)
T × PaR	1	1.82	0.18	1.39	0.24
V × PaR	3	17.57	<0.001	17.14	<0.001

**Table 6:** Log linear results showing the effect of each factor (T = treatment; V = vineyard) on predation rate (Pr = predation rate).

Effect	df	Chi Square (Partial Association Test)	*p* (Partial Association Test)	Chi Square (Marginal Association Test)	*p* (Marginal Association Test)
T × Pr	1	12.46	<0.001	37.96	<0.001
V × Pr	3	188.44	<0.001	213.94	<0.001

## Supplementary Materials

The following are available online at https://www.mdpi.com/article/10.3390/insects12040330/s1. Figure S1: Percentage of infested bunches (± binomial SE) in sugar dispenser and control plots in vineyard 1, vineyard 2, vineyard 3 and vineyard 4, Figure S2: Parasitization rate on colonies (± binomial SE) in sugar dispenser and control treatments in vineyard 1, vineyard 2, vineyard 3 and vineyard 4, Figure S3: Percentage of parasitized mealybugs (± binomial SE) on randomly-collected bunches in sugar dispenser and control plots in vineyard 1, vineyard 2, vineyard 3 and vineyard 4, Figure S4: Percentage of predated mealybugs on colonies (± binomial SE) in sugar dispenser and control treatments in vineyard 1, vineyard 2, vineyard 3 and vineyard 4.
