# Supplementary material for: Use of Sugar Dispensers to Disrupt Ant Attendance and Improve Biological Control of Mealybugs in Vineyard
Source: Insects. 2021 Apr 7;12(4):330. doi: 10.3390/insects12040330 (PMC8068082; doi:10.3390/insects12040330)
Supplement: Supplementary file 1 [file insects-12-00330-s001.pdf]

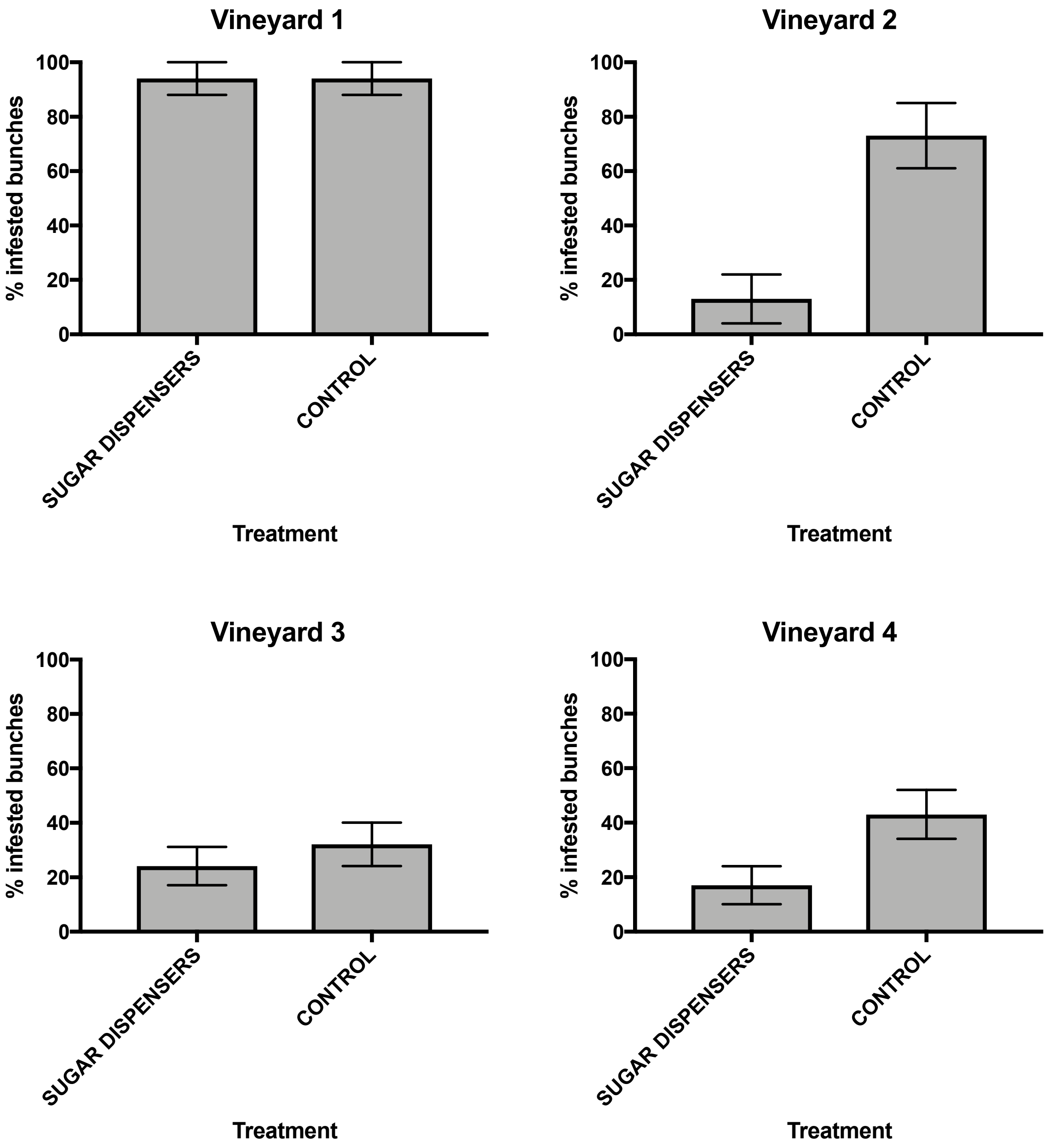

Figure S1. Percentage of infested bunches (± binomial SE) in sugar dispenser and control plots in vineyard 1, vineyard 2, vineyard 3 and vineyard 4.

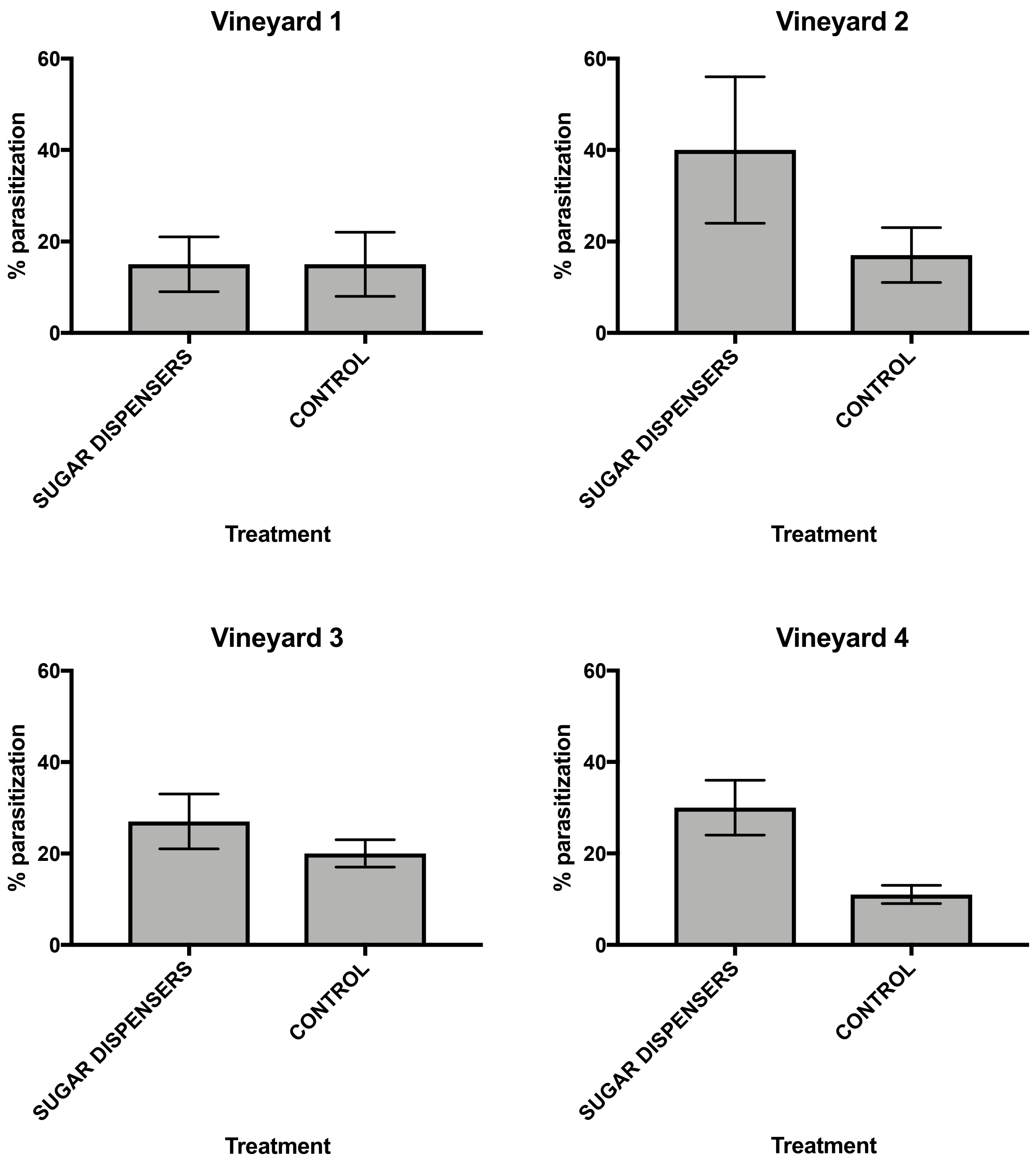

Figure S2. Parasitization rate on colonies (± binomial SE) in sugar dispenser and control treatments in vineyard 1, vineyard 2, vineyard 3 and vineyard 4.

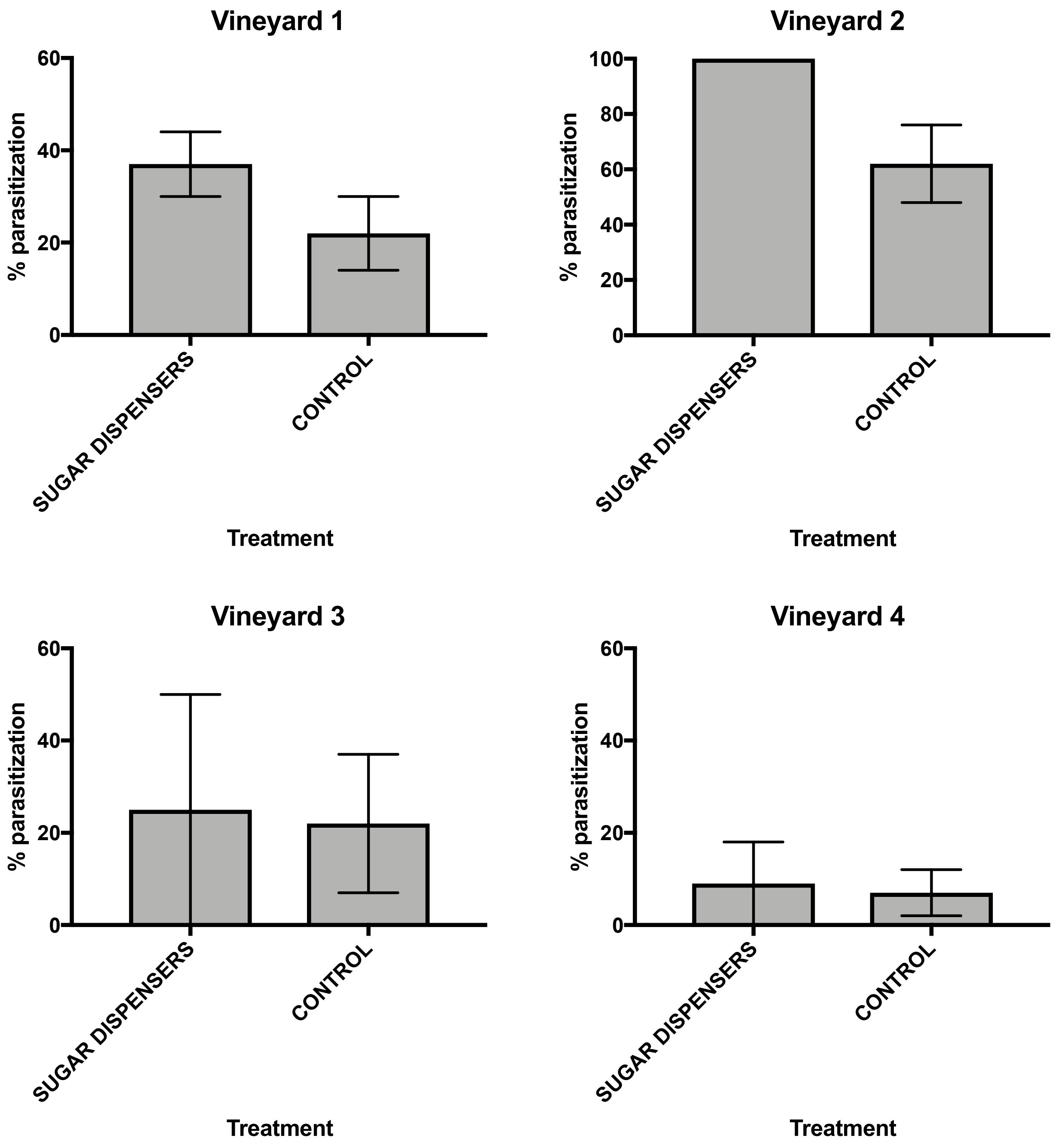

Figure S3. Percentage of parasitized mealybugs (± binomial SE) on randomly-collected bunches in sugar dispenser and control plots in vineyard 1, vineyard 2, vineyard 3 and vineyard 4.

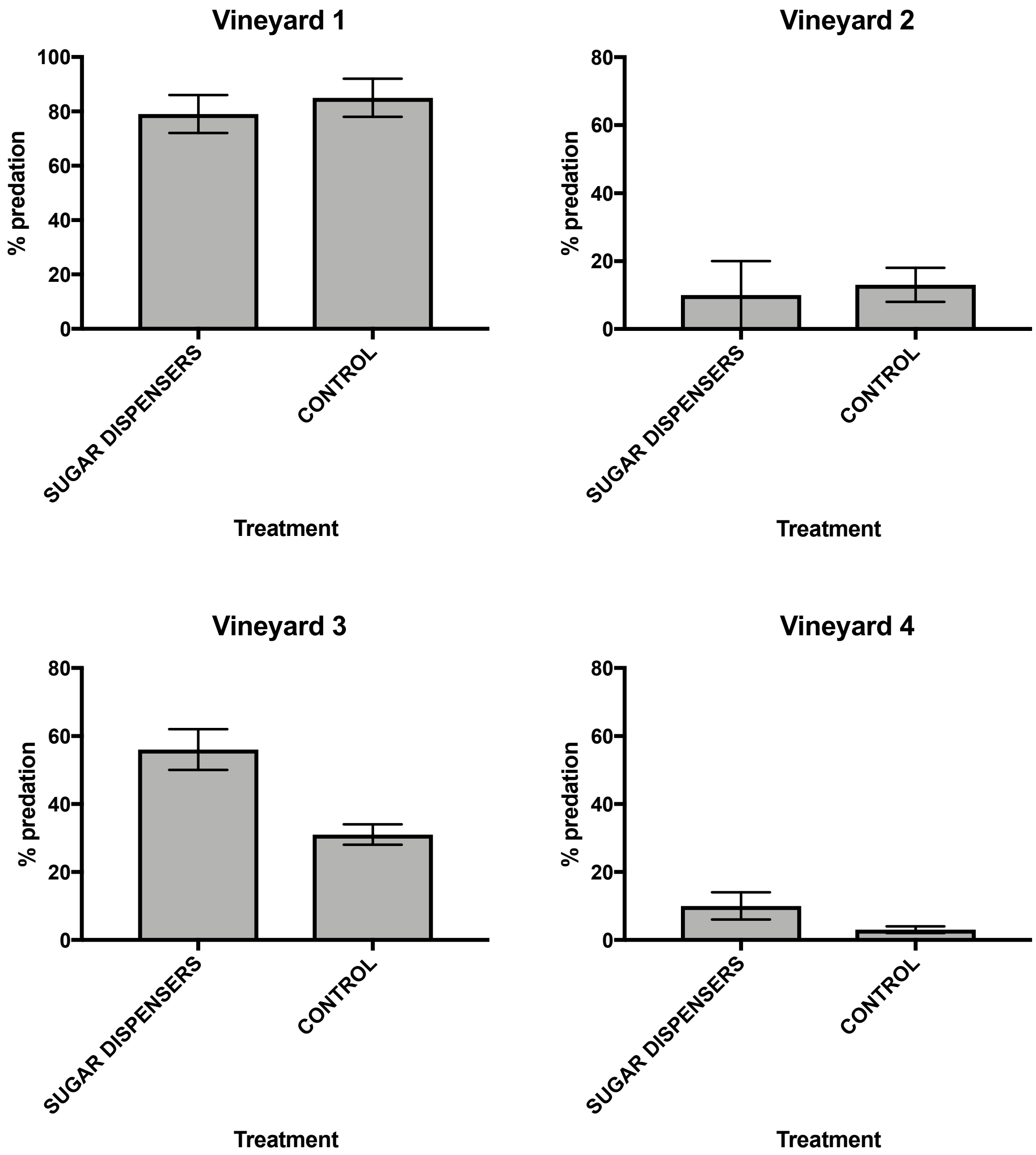

Figure S4. Percentage of predated mealybugs on colonies (± binomial SE) in sugar dispenser and control treatments in vineyard 1, vineyard 2, vineyard 3 and vineyard 4.
